# New Insights into the Role of miR-29a in Hepatocellular Carcinoma: Implications in Mechanisms and Theragnostics

**DOI:** 10.3390/jpm11030219

**Published:** 2021-03-18

**Authors:** Ya-Ling Yang, Yen-Hsiang Chang, Chia-Jung Li, Ying-Hsien Huang, Ming-Chao Tsai, Pei-Yi Chu, Hung-Yu Lin

**Affiliations:** 1 Department of Anesthesiology, Kaohsiung Chang Gung Memorial Hospital and Chang Gung University College of Medicine, Kaohsiung 833, Taiwan; inr453@cgmh.org.tw; 2 Department of Nuclear Medicine, Kaohsiung Chang Gung Memorial Hospital and Chang Gung University College of Medicine, Kaohsiung 833, Taiwan; changyh@cgmh.org.tw; 3 Center for Mitochondrial Research and Medicine, Kaohsiung Chang Gung Memorial Hospital, Kaohsiung 833, Taiwan; 4 Department of Obstetrics and Gynecology, Kaohsiung Veterans General Hospital, Kaohsiung 813, Taiwan; nigel6761@gmail.com; 5 Department of Pediatrics, Kaohsiung Chang Gung Memorial Hospital and Chang Gung University College of Medicine, Kaohsiung 833, Taiwan; yhhuang123@yahoo.com.tw; 6 Division of Hepato-Gastroenterology, Department of Internal Medicine, Kaohsiung Chang Gung Memorial Hospital and Chang Gung University College of Medicine, Kaohsiung 833, Taiwan; tony0779@gmail.com; 7 Department of Pathology, Show Chwan Memorial Hospital, Changhua 500, Taiwan; 8 School of Medicine, College of Medicine, Fu Jen Catholic University, New Taipei City 242, Taiwan; 9 Department of Health Food, Chung Chou University of Science and Technology, Changhua 510, Taiwan; 10 National Institute of Cancer Research, National Health Research Institutes, Tainan 704, Taiwan; 11 Research Assistant Center, Show Chwan Memorial Hospital, Changhua 500, Taiwan

**Keywords:** hepatocellular carcinoma, miR-29a, diagnosis, therapeutics, carcinogenesis, epigenetics, metabolic adaptation, competitive endogenous RNAs, tumor microenvironment, fibrosis, metastasis, angiogenesis, immunomodulation

## Abstract

Hepatocellular carcinoma (HCC) remains one of the most lethal human cancer globally. For advanced HCC, curable plan for advanced HCC is yet to be established, and the prognosis remains poor. The detail mechanisms underlying the progression of HCC tumorigenicity and the corruption of tumor microenvironment (TME) is complex and inconclusive. A growing body of studies demonstrate microRNAs (miRs) are important regulators in the tumorigenicity and TME development. Notably, mounting evidences indicate miR-29a play a crucial role in exerting hepatoprotective effect on various types of stress and involved in the progression of HCC, which elucidates their potential theragnostic implications. In this review, we reviewed the advanced insights into the detail mechanisms by which miR-29a dictates carcinogenesis, epigenetic program, and metabolic adaptation, and implicated in the sponging activity of competitive endogenous RNAs (ceRNA) and the TME components in the scenario of HCC. Furthermore, we highlighted its clinical significance in diagnosis and prognosis, as well as the emerging therapeutics centered on the activation of miR-29a.

## 1. Introduction

Liver cancer stands at the fourth leading cause of cancer-related deaths globally, with approximately 850,000 new cases and 780,000 deaths occurred in 2018 [1,2]. Hepatocellular carcinoma (HCC) is characterized by easy metastasis and poor prognosis, accounting for about 80% of primary liver cancers [2]. The risk factors contributing to hepatocarcinogenesis include hepatitis B virus (HBV) and hepatitis C virus (HCV), alcoholic liver disease (ALD), nonalcoholic steatohepatitis (NASH), and nonalcoholic fatty liver disease (NAFLD) [3]. For HCC in early stage, a comprehensive treatment plan mainly employs surgical resection and combined with transcatheter arterial chemoembolization (TACE) and radiofrequency ablation [4]. However, most of the HCC cases have been exacerbated to an advanced stage at the first diagnosis, losing the optimal opportunity for surgical resection. In terms of advanced HCC, no curable plan is established, and the five-year survival rate stands at 3~5% [5]. The regrettable outcome can be attributable to late diagnosis, resistance to anti-cancer treatment, and high frequency of recurrence [6,7]. Thus, elucidation of the underlying mechanism and pathogenesis of HCC is crucial for paving the way to develop new diagnostic/prognostic biomarkers and therapeutic approaches.

MicroRNAs (miRs) are a class of small RNAs composed of 18~22 nucleotides in length and known as powerful regulators of gene expression [8]. In the nucleus, RNA polymerase II/III initially transcribes primary miRs (pri-miRs), which will be subsequently processed into a ~70-nt precursor miRs (pre-miRs) by microprocessor complex comprising an endoribonuclease Drosha and a double stranded-RNA binding protein DiGeorge syndrome critical region 8 (DGCR8) [9]. Pre-miRs are then exported into cytoplasm through an exportin 5/guanosine 5′-triphosphate (XPO5/RanGTP) complex. In the cytoplasm, endonuclease Dicer cleaves the loop of a pre-miRNA, leading to a ~22-nt mature miR [10]. Then one strand of mature miR (passenger strand) is degraded, while the other strand (guide strand or miRNA) is engaged with the Argonaut proteins (AGOs) to form the RNA-induced silencing complex known as RISC [11]. Following its association within RISC, a 6~8-mer sequence of miR, known as a seed sequence exerts its function by complementarily binding to a strand of mRNA on its 3′ untranslated region (3′UTR) and negatively regulates mRNA expression in most cases [12]. 

**Figure 1:**
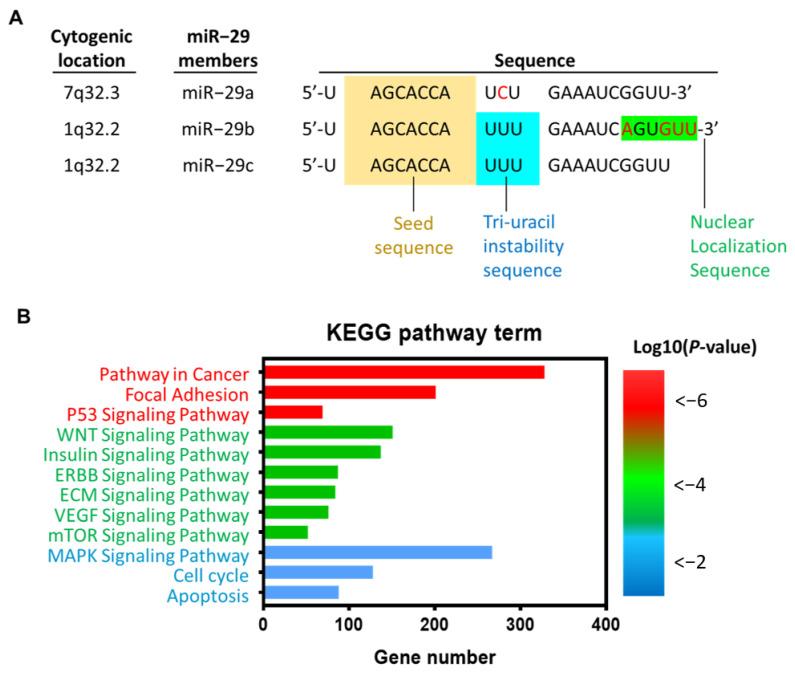
Genetic properties and biological functions of miR-29. (**A**) Schematic illustration of the miR-29a family members. (**B**) The enrichment analysis of miR-29 targets in Kyoto Encyclopedia of Genes and Genomes (KEGG) pathways was conducted by ENCORI (The Encyclopedia of RNA Interactomes). The histogram demonstrates number of miR-29a-targeted genes involved in particular KEGG pathway terms. The log10(*p*-value) of each KEGG terms is expressed by a heatmap.

miR-29 family consists of three members, miR-29a, miR-29b, and miR-29c. In human, miR-29a and miR-29b-1 are encoded in chromosome 7q32.3, while miR-29b-2 and miR-29c are encoded in chromosome 1q32.2 (Figure 1A). miR-29 family members share common 7-mer seed sequence. Different from miR-29a and -29c, miR-29b harbors a unique 6-nt segment at position 18–23 (Figure 1A), rendering miR-29b a transferable nuclear localization element [13]. Furthermore, miR-29a has one notable difference from miR-29b and -29c at position 10, which is a distinct cytosine residue forming a UCU sequence at position 9–11 (Figure 1A). Unlike miR-29a, miR-29b and miR-29c harbor a tri-uracil sequence (UUU) at position 9–11, which results in instability of nucleotides and thereby rapid decay [14]. With regard to clinical implication, we surveyed the KEGG pathways of miR-29 by suing ENCORI (The Encyclopedia of RNA Interactomes) [15], and noted that the biological functions are predominantly involved in cancer progression and signaling pathway dictating tumorigenicity (Figure 1B).

miR-29a has been demonstrated to have an important role in a multitude of pathophysiological processes, ranging from neurodegenerative diseases [16,17,18], cardiovascular diseases [19,20,21], pulmonary disorders [22,23], tendon disease [24], renal fibrosis [25], and scleroderma [26], and liver disease [27,28]. The hepatoprotective effect of miR-29a in the context of acute cholestasis and chronic diet stress has notably been demonstrated in our previous studies [29,30,31,32,33,34,35,36,37,38,39,40]. More importantly, a growing body of study has revealed the key role of miR-29a in the pathogenesis of HCC and its potential as a theragnostic target is notably emerging. This promising progress prompted us to review the advanced insights into miR-29a in the progression of HCC and its impact on the formation of tumor microenvironment (TME).

## 2. miR-29a in Carcinogenesis

Carcinogenesis is often initiated with cells exhibiting uncontrolled proliferation after being subjected to accumulated mutations [41]. This phenomenon usually involves the dysregulation of oncogenes, tumor suppressor genes, and those required for DNA repair mechanism [41]. As such, additional mutations and disturbed epigenetic activity contributes to the clonal selection with more aggressive phenotypes. In this regard, hepatitis B virus (HBV) is a critical hepatocarcinogenic factor through inducing mutations and therefore dysregulation of important genes, including tumor suppressing gene p53 and phosphatase and tensin homolog (PTEN), as well as genes responsible for anti-apoptosis and cell cycle progression [42]. Furthermore, the presence cancer stem cell (CSC) could bolster hepatocarcinogenesis due to their biological hallmarks including self-renewal, apoptotic resistance, and drug resistance [41].

There are some reports suggesting that miR-29a play a positive role in fostering carcinogenesis. Kong et al. have reported that hepatitis B virus (HBV) X protein (HBx)-induced hepatocarcinogenesis is mediated by miR-29a, wherein the overexpression of miR-29a promoted the migration of HepG2 cells [43]; while miR-29a inhibitor abolished the enhanced migratory ability of HBx-transfected hepatoma cells. Mechanistically, miR-29a was identified to suppress PTEN by binding to its 3′UTR, thereby augmenting the AKT activity that relays cancer progression [43]. In addition, Wu et al. demonstrated that miR-29a acts to promote HBV replication in HBV-expressing cells by inhibiting the repressor of HBV replication, SWI/SNF-related matrix-associated actin-dependent regulator of chromatin subfamily E member 1 (SMARCE1) [44].

**Figure 2:**
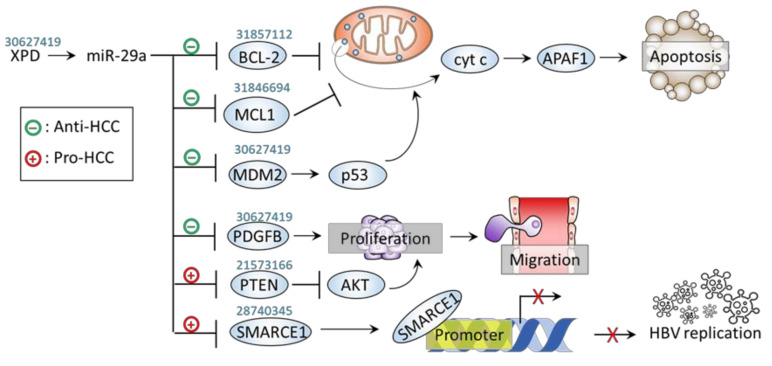
miR-29a and HCC carcinogenesis. Graphic illustration of miR-29a signaling pathway on carcinogenic factors. The PubMed identifier (PMID) of references of interest are indicated above the corresponding genes.

In contrast to the tumor-promotive role of miR-29a reported in the aforementioned context of HBV-related HCC, a couple of studies have indicated its tumor-suppressive role on carcinogenesis through activation of apoptotic pathway. Xiao et al. indicated a counteracting role of miR-29a on carcinogenesis of HCC [45]. In this regard, Xiao et al. demonstrated that miR-29a acts to inhibit oncogene mouse double minute 2 (MDM2), leading to restored levels of tumor suppressor p53. In addition to MDM2, miR-29a was shown to suppress the other oncogene platelet-derived growth factor subunit B (PDGFB) [45]. In this context, a subunit of transcription factor II H (TFIIH), xeroderma pigmentosum D (XPD), was revealed to upregulate miR-29a expression levels, thereby counteracting HCC cell proliferation and migration in vitro and xenograft tumor growth in vivo [45]. Song et al. demonstrated that miR-29a acts as a suppressor of CSC [46]. Overexpression of miR-29a inhibited self-renewal and tumorigenesis as well as potentiate cytotoxicity of sorafenib by downregulating BCL-2 via binding its 3’UTR, while miR-29a knockdown showed a reversed effect [46]. Moreover, the therapeutic and sorafenib-sensitizing effect of miR-29a was recaptured in patient-derived xenografts (PDXs) experimental model [46]. In addition, a study reported by Li et al. support the notion that miR-29a-mediated suppression on anti-apoptotic factor acts to impede HCC progression. In this regard, mutant ATCB mRNA exerts sponging activity to downregulate miR-29a levels, thereby unleashing its suppression on the anti-apoptotic factor MCL1 and leading to enhanced HCC tumorigenicity, suggesting miR-29a’s negative role in promoting HCC apoptosis [47]. Taken together, we noted that miR-29a exerts tumor-promotive effect in the scenario of HCC involved in HBV infection, whereas it acts to activate apoptotic pathway to mediate tumor-suppressive effect in non-HBV contexts of HCC. Further study should be conducted to decipher the molecular basis that underlies the contradictory conditions. The roles and mechanisms of miR-29a on hepatocarcinogenesis are listed and illustrated in Table 1 and Figure 2.

## 3. miR-29a as an Epigenetic Regulator

**Figure 3:**
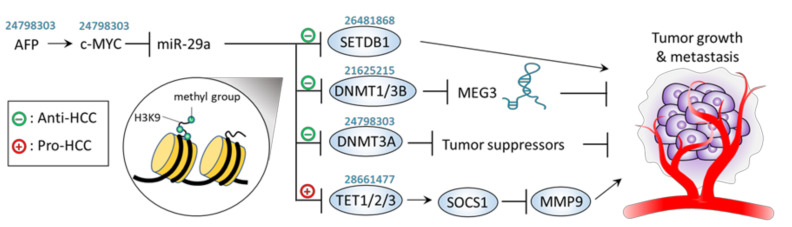
miR-29a as an epigenetic modifier. Graphic illustration of miR-29a signaling pathway on epigenetics in the context of tumor growth and metastasis. The PMID of references of interest are indicated above the corresponding genes.

miR-29a acts to target epigenetic modifiers in HCC cells, wherein most reports identified its negative role in tumor progression. Braconi et al. showed that miR-29a acts as a negative regulator on DNA methyltransferase (DNMT)1 and DNMT3B in HCC cells, leading to hypomethylated promoter of long non-coding RNA maternally expressed 3 (MEG3) [48]. The resultant increase in MEG3 levels exerts suppressive effect on HCC cell growth [48]. Parpart et al. have demonstrated that HCC biomarker alpha-fetoprotein (AFP) acts to mediate tumor growth by regulating c-MYC/miR-29a axis. Specifically, overexpressed AFP activated c-MYC to bind to the miR-29a promoter and to the intron in the presence of AFP, as evidenced by CHIP assay. This AFP/c-MYC/miR-29a-centric epigenetic control then plays a factor in increased levels of DNMT3A and DNMT3B which may inhibit tumor suppressor gene expression and drive aggressive HCC and poor outcome [49]. Similarly, Kogure et al. found that miR-29a inhibited DNMT1 and DNMT3B to counteract the increased expression levels of EMT marker E-cadherin induced by TGF-β in PLC/PRF/5 hepatoma cells [50]. Wong et al. identified that miR-29a negatively regulates the oncogenic histone methyltransferase, SET domain, bifurcated 1 (SETDB1) through direct interaction at post-transcriptional level. Down-regulated levels of SETDB1 predicted good outcome and functionally impeded HCC cell proliferation and migration in vitro and HCC tumorigenicity in lung metastasis in vivo [51]. Gao et al. exhibited that miR-29a exerts inhibitory effect on HCC progression by down-regulating histone lysine N methyltransferase 5C (KMT5C), which acts as an HCC biomarker and prognostic indicator and functions as an HCC oncogene [52]. miR-29a acted as a mandatory factor to mediate anti-HCC effect derived from knockdown of KMT5C [52]. Furthermore, circMYLK was identified as a negative regulator of miR-29a through competitive sponging mechanism [52]. In contrast, a study reported by Chen et al. showed that miR-29a acts toward epigenetic modification to exert a pro-tumor effect [53]. In this regard, miR-29a was identified to inhibit critical DNA methylation modifiers, ten eleven translocation (TET) family members. By virtue of this activity, overexpression of miR-29a caused a decrease in anti-metastatic suppressor of cytokine signaling 1 (SOCS1) an increase in pro-metastatic matrix metalloproteinase 9 (MMP9), ultimately resulting in enhanced proliferation, migration, and invasion of HCC cells in vitro and tumor growth and metastasis in vivo [53]. Herein we summarized miR-29a’s roles as an epigenetic regulator in Table 2 and Figure 3.

## 4. miR-29a Acts to Counteract Metabolic Adaptation

**Figure 4:**
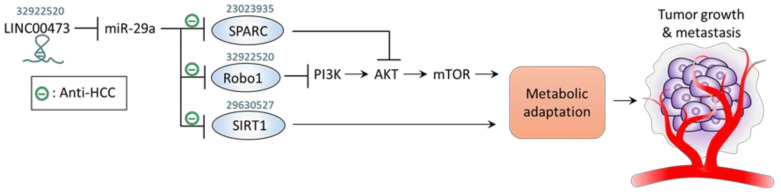
Role and mechanism of miR-29a in metabolic adaptation. Graphic illustration of miR-29a signaling pathway on metabolic adaptation in the context of tumor growth and metastasis. The PMID of references of interest are indicated above the corresponding genes.

Metabolic adaptation is a central factor that contributes to ensuring the supply of cellular building blocks including proteins, lipids, and nucleotides in order to maintain a high proliferative rate of HCC [54]. In this regards, miR-29a has been reported to act toward genes involved in metabolism to impede HCC progression. Zhu et al. revealed that miR-29a levels are reduced in HCC tissue compared to adjacent normal tissue and in HCC cell lines compared to normal liver cell line [55]. Overexpression of miR-29a suppressed proliferation of HCC cells by direct targeting secreted multi-functional matricellular glycoprotein and rich in cysteine (SPARC), wherein inhibited SPARC led to the inactivation of its downstream effector AKT and ultimately reduced activity of mammalian target of rapamycin (mTOR) and extracellular signal-regulated kinase (ERK) [55]. Zhang et al. demonstrated that HCC tissue/cells express higher levels of SIRT1, which can act as cellular regulator for accommodating metabolic stress, and simultaneously lower levels of miR-29a. Further validation approach confirmed that miR-29a directly targets SIRT1 to inhibit the proliferation and cell cycle progression of HCC [56]. Li et al. showed that overexpression of miR-29a suppressed glycolytic phenotype to impede proliferation, migration, and invasion of HCC by targeting guanylyl cyclase domain containing 1 (GUCD1) [57]. In addition, the role of circRNA circ-ZNF652 in positively regulating miR-29a expression levels in HCC tumorigenicity was revealed [57]. More recently, Song et al. demonstrated that miR-29a exerts HCC-counteracting effect by suppressing oncogene roundabout homolog 1 (Robo1) with direct binding at post-transcriptional level [58]. Downregulated Robo1 by lentivirus-based miR-29a overexpression is involved in the inactivation of PI3K/AKT/mTOR axis, leading to impeded HCC cell proliferation, migration, and invasion in vitro and alleviated HCC tumor growth and metastasis in vivo [58]. Furthermore, lncRNA LINC00473 was identified to be a negative regulator of miR-29a through a competitive sponging mechanism, thereby acting toward a pro-tumor effect [58]. The anti-HCC effect and corresponding pathways mediated by miR-29a are shown in Table 3 and Figure 4.

## 5. Molecular Sponges Targeting miR-29a: ceRNAs

**Figure 5:**
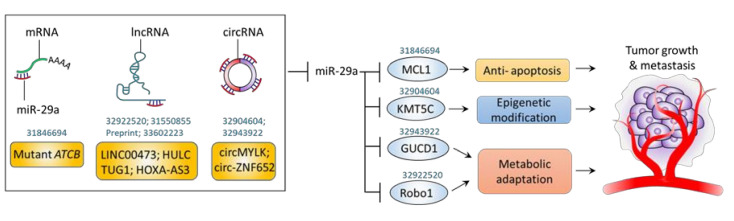
ceRNA acts as miR-29a sponge to positively promote HCC progression. Graphic illustration of ceRNAs-mediated suppression of miR-29a and its downstream signaling pathway involved in tumor growth and metastasis. The PMID of references of interest are indicated above the corresponding genes.

ceRNAs are specific RNAs that can impede miR activity through competitively sponging a particular pool of miRs at miR recognition element (MRE), thereby resulting in the upregulation of miR target gene [59]. A variety of RNAs regulate miRs expression levels by the miR sponging mechanism, including long non-coding RNAs (lncRNAs), circular RNAs (circRNAs), protein-coding transcripts (mRNAs) pseudogene transcripts, and expressed 3′UTR [59]. More recently, several ceRNAs that sponge miR-29a in the context of HCC have been recognized. Li et al. demonstrated that mutant β-actin (ACTB) mRNA 3′-UTR promotes HCC proliferation, migration, and invasion in vitro and tumor growth in vivo by regulating miR-29a in an argonaute RISC catalytic component 2 (AGO2)-dependent manner [47]. Identified as most frequently mutated housekeeping gene in HCC tissue, mutant ACTB 3′UTR acted to inhibit miR-29a by direct interaction, whereby miR-29a target gene MCL1 apoptosis regulator, BCL2 family member (MCL1) was up-regulated to bolster tumor progression [47]. Song et al. reported that lncRNA LINC00473 plays a promotive role in HCC by competitive sponging miR-29a [58]. shRNA-based knockdown of LINC00473 led to an increased level of miR-29a and thereby a down-regulated Robo1. The reduced Robo1 is involved in the inactivation of PI3K/AKT/mTOR axis, leading to an impeded HCC cell proliferation, migration, and invasion in vitro and HCC tumor growth and metastasis in vivo [58]. Zhu et al. showed that lncRNA HULC promotes HCC cells proliferation by functioning to sponge and downregulate miR-29 [60]. A preprint report conducted by Liu et al. showed that lncRNA TUG1 competitively targets miR-29a to upregulate the expression of a oncogenic factor, interferon-induced transmembrane protein 3 (IFITM3), resulting in increased proliferation, invasion, and migration of HCC [61]. In addition, Qu et al. identified that lncRNA HOXA-AS3 acts as a tumor-promoting factor in gastric cancer cells by suppressing miR-29a, leading to the activation of lymphotoxin β receptor (LTBR)/NF-κB signaling [62]. Gao et al. unraveled that a circMYLK exerted HCC-promoting effect by serving as a ceRNA of miR-29a, whereby oncogene histone lysine N methyltransferase 5C (KMT5C) can be upregulated [52]. siRNA knockdown of circMYLK restored the miR-29a expression levels, leading to the downregulation of KMT5C. The decreased KMT5C expression level acted toward impeded tumoral behavior of HCC [52]. Li et al. reported that circ-ZNF652 acts as molecular sponge to miR-29a, thereby positively upregulating the GUCD1 [57]. shRNA-knockdown of circ-ZNF652 served as a therapeutic approach to restore the miR-29a/GUCD1 axis activity, leading to a suppressed tumorigenic behavior in vitro and in vivo [57]. Table 4 and Figure 5 illustrate the recent insights into ceRNA-mediated sponging mechanisms on suppressing the activity of miR-29a, thereby leading to a tumor-promoting effect in the context of HCC.

## 6. Inhibitory Effect of miR-29a on Tumor Microenvironment Components

**Figure 6:**
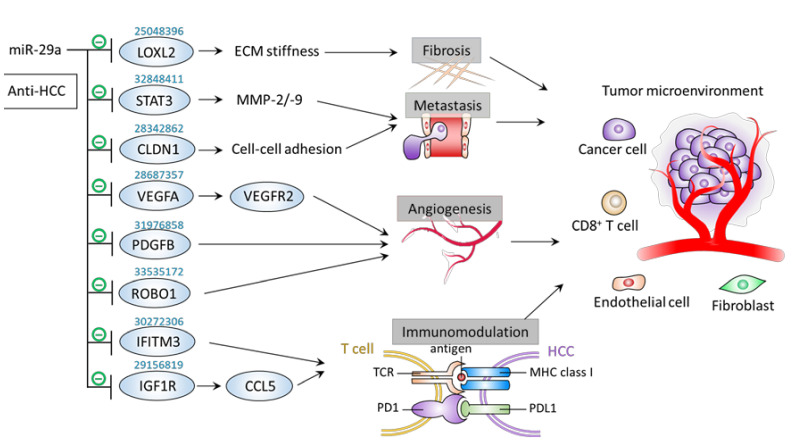
miR-29a as a regulator of tumor microenvironment. Graphic illustration of miR-29a signaling pathway on factors contributing to the corruption of tumor microenvironment. The PMID of references of interest are indicated above the corresponding genes. The PMID of references of interest are indicated above the corresponding genes.

Tumor cells communicate with the surrounding microenvironment components implicated in tumor metastasis, ECM stiffness or fibrosis, angiogenesis, and immunomodulation to level up their ability of growth and invasiveness. In this regard, the role of a cluster of miRs in relaying the crosstalk between HCC and its microenvironment has been extensively reviewed [63]. Herein we highlighted miR-29a’s pathway-centric manner in dictating the tumor microenvironment (TME) remodeling to impede various biological factors bolstering tumor progression (Table 5 and Figure 6).

### 6.1. Metastasis

Claudin 1 (CLDN1) is involved in cell–cell connection and regarded as an HCC oncogene due to its role in promoting EMT phenotype acquisition [72]. Mahati et al. revealed that tumor tissue levels of miR-29a inhibit CLDN1 by directly binding to its 3′UTR [66]. Notably, overexpression of miR-29a acted to reduced HCC cell proliferation and migration in vitro and tumor growth in vivo in a CLDN1-dependent manner [66]. Signal transducer and activator of transcription 3 (STAT3) overexpression serves as a poor prognostic factor and functionally plays a positive role in promoting HCC progression by critical molecular events, including acquisition of EMT phenotype and increased expression levels of matrix metalloproteinase (MMP)-2 and -9 [73]. STAT3 as a therapeutic target has attracted attention and notably tested efficaciously [73]. More recently, Shi et al. demonstrated that miR-29a presents a negative correlation with STAT3 in HCC tumor tissue [65]. Functionally, miR-29a downregulated STAT3 expression levels by direct binding at post-transcriptional level, leading to an inhibitory effect on HCC cell proliferation, migration, and invasion [65]. Interestingly, a specified prescription of traditional Chinese medicine “Xiaoai Jiedu Recipe” was identified to mediate anti-HCC effect by virtue of the miR-29a/STAT3 axis activation [65].

### 6.2. Fibrosis

Fibrosis is mediated by activated cancer-associated fibroblasts and is a complex process that involves excessive deposition and reorganization of the extracellular matrix (ECM), leading to epithelial-to-mesenchymal transition (EMT) reprogramming. For example, a remodeled, stiff ECM can support angiogenesis with abnormal vasculature, leading to a corrupt TME which promotes tumor dissemination [74]. In this regard, the function of lysyl oxidase (LOX) family members on a pro-tumoral TME of HCC by remodeling the ECM was recently noted [75]. More notably, Wong et al. demonstrated that miR-29a acts as a negative regulator on lysyl oxidase like 2 (LOXL2) through interacting with the 3’UTR, thereby impeding the HCC metastasis [64].

### 6.3. Angiogenesis

Angiogenesis is a hallmark of HCC that is necessary for tumor growth and progression [76,77]. Hypoxic tumor microenvironment in HCC plays a factor to promote angiogenesis by upregulating hypoxia-inducible signaling pathways, which can be a promising drug target [78]. For example, the vascular endothelial growth factor/vascular endothelial growth factor receptor (VEGF/VEGFR) pathway has been validated as a drug target in HCC [79,80]. Although the role of miR-29a in the context of HCC angiogenesis is still lacking, some reports have shown its counteracting effect on angiogenic activity in the scenario of other types of cancer. Zhong et al. demonstrated that miR-29a inhibited VEGFA by directly targeting to its 3′UTR, thereby hindering the VEGFA/VEGFR2 pathway to exert tumor-suppressive effect in bladder carcinoma [69]. Furthermore, Rosano et al. demonstrated that miR-29a plays a negative role in inhibiting VEGFA-induced sprouting process of endothelial cells and that an upregulation of genes that are targeted by miR-29a during sprouting angiogenesis correlates with tumor angiogenesis in samples of colorectal cancer patients [68]. More recently, Zhang el al. revealed an inhibitory role of miR-29a on migration and vasculogenic mimicry formation in glioma cell through direct 3′UTR binding and thereby suppressing the expression of ROBO1 [67]. Nevertheless, more studies are warranted to gain more insights into the effect of miR-29a on HCC angiogenesis as well as its detail molecular mechanisms.

### 6.4. Immunomodulation

miR-29a has been shown to play a role in mediating immune signaling in the microenvironment of hematologic malignancy [81]. Although miR-29a has not been scrutinized intensively with respect to the immunity of HCC, a few studies have started to reveal its emerging role in this context.

Liang et al. have reported that IFITM3, an immunosuppressive factor [82], exhibits positive effect on HCC proliferation, migration, and invasion [70]. Of note, miR-29a was identified to directly target IFITM3, thereby counteracting the invasive phenotypes of HCC [70]. A study conducted by Wang et al. reported the anti-HCC effect of miR-29a is implicated in the immunomodulation on the interaction between HCC cells and immune cells [71]. Overexpression of miR-29a down-regulated insulin-like growth factor 1 receptor (IGF1R) to restrict growth, migration, and invasion of HCC cell by targeting IGF1R 3′UTR [71]. HCC with down-regulated IGF1R led to an increase of C-C motif chemokine ligand 5 (CCL5) secretion, which may function as a chemotactic factor for immune cell in a tumor microenvironment. Indeed, supernatant from IGF1R-downregulating HCC boosted migration of CD8+ T lymphocytes in a transwell-based chemotaxis assay compared to that from corresponding control cells, indicating that miR-29a acts as a tumor suppression gene and a potential target for improving immunosuppressive status in tumor microenvironment of HCC [71] and thereby may serve as a candidate to advance the efficacy of immunotherapy. Interestingly, Xu et al. reported that miR-29a targets immunoinhibitory molecule B7-H3 in HeLa cell and inhibits B7-H3 expression levels [83], implying the potential of miR-29a in potentiating immunotherapy. However, the exact molecular mechanism underlying how miR-29a affects adaptive immune resistance mechanism, e.g., interaction between PD-1 and PD-L1 and TCR-centric signaling, by which cancer cells evade the host immune response in the context of HCC requires further study to elucidate.

## 7. miR-29a as a Diagnostic/Prognostic Indicator

The advanced insights into the predictive and prognostic relevance of miR-29a in HCC patients are summarized in Table 6. There are several studies reported that decreased expression levels of miR-29a act as a biomarker of HCC tissue [45,53,55,56,65,66,70,84,85]. Parpart et al. have identified an inverse correlation between miR-29a and AFP where tumor miR-29a level decrease significantly as serum AFP levels increase. In addition, low levels of miR-29a predict poor OS [49]. Similarly, Mahati et al. demonstrated that low levels of miR-29a in HCC tumor tissue predict poor OS [66]. Liang et al. demonstrated the predictive value of low miR-29a in larger tumor size (>5 cm), multifocal sites, venous invasion, and poor overall survival (OS) [70]. Zhang et al. reported similar observations and additionally noted that low levels of miR-29a predict poor disease-free survival (DFS) [56]. Li et al. reported that decreased miR-29a levels are associated with lymph node metastasis and late TNM stage (III-IV) [65]. On the contrary, a few reports revealed that an increase in miR-29a levels of HCC tissue is associated with poor outcome. In this regard, Chen et al. showed that increased miR-29a levels predict poor OS [53]. Zhu et al. have revealed that elevated miR-29a levels of tumor tissue served as an independent predictor for early recurrence, and short OS time in HBV-related HCC after surgical resection [86].

In terms of clinicopathological feature of circulating miR-29a, we noted that a couple of reports indicated increased circulating miR-29a levels are associated with poor outcome, whereas one study revealed decreased circulating miR-29a levels as a prognostic indicator. Zhu et al. demonstrated that increased serum miR-29a levels can serve as not only a differential biomarker for HBV-positive HCC, but also a predictor for poor OS and progression-free survival (PFS) [88]. Lin et al. demonstrated that that a serum-based miR-classifier containing increased miR-29a, along with miR-29c, miR-133a, miR-143, miR-145, miR-192, and miR-505, exhibits higher sensitivity than and similar specificity to α-fetoprotein (AFP) in detecting HCC at the time of diagnosis. Furthermore, the miR-classifier presented larger area under the receiver operating characteristic curve (AUC) than did α-fetoprotein at a cutoff of 20 ng/mL (AFP20) with respect to identifying small-size, early-stage, and AFP-negative HCC [89]. Xue et al. demonstrated that a cluster of serum exosomal miRs of HCC patients, including miR-29a as well as miR-122, miR-125b, miR-145, miR-192, miR-194, miR-17-5p, and miR-106a is significantly higher than that of normal controls, indicating a potential diagnostic panel for HCC [90]. In contrast, Cho et al. showed that decreased plasma miR-29a levels independently predict poor DFS and poor liver-transplantation-free survival rate [87]. 

## 8. Therapeutic Approaches Targeting miR-29a for the Treatment of HCC

Most of the reviewed studies indicate suppressive role of miR-29a in suppressing the HCC tumorigenicity. Indeed, we noted that mounting evidences reveal that therapeutics acting toward the upregulation of miR-29a present significant efficacy (Table 7). Song et al. reported that sevoflurane, an anesthetic drug, induces the expression level of miR-29a and accordingly inhibited the corresponding target DNMT3A, leading to pro-apoptotic effect on HCC [84]. Zamani et al. utilized dendrosome nanoparticle to carry curcumin (dendrosomal curcumin, DNC) in an attempt to enhancing its solubility and hence the anti-cancer effect to HCC [91]. They showed that DNC effectively penetrates into HepG2 and Huh-7 cells and thereby significantly reduces the cell viability. Mechanistically, DNC induced an overexpression of miR-29a/miR-185 which acts to down-regulate the expression of DNMT1/3A/3B. The resultant alteration in the promoter hypomethylation of tumor-suppressive long non-coding RNA maternally expressed 3 (MEG3) led to an overexpression of MEG3 [91]. Meng et al. showed that arsenic trioxide (As2O3), an approved drug for leukemia treatment, exerts pro-apoptosis effect through a miR-29a-dependent manner in HepG2 hepatoma cells [92]. Furthermore, a meta-analysis conducted by He et al. indicated that the combination of As_2_O_3_ and TACE acts toward decreased AFP levels, increased one-year survival rate, improved patients’ quality of life, and attenuated the side effects of chemotherapy with only adverse events of mild to moderate grades reported [93]. Specifically, arsenic trioxide-induced miR-29a acted to inhibit protein phosphatase Mg^2+^/Mn^2+^-dependent 1D (PPM1D), an anti-apoptosis gene involved in p53-replated pathway, leading to cytotoxicity [92]. Shi et al. revealed that traditional Chinese medicine Xiaoai Jiedu Recipe (XJP) boosts miR-29a expression levels to suppress HCC tumorigenicity in a dose-dependent manner. The effect of XJP is mediated by the activity of miR-29a on inhibiting STAT3, leading to a counteracted tumor growth and invasiveness [65]. More recently, targeting miRNA sponging mechanism for the upregulation of miR-29a provides an emerging strategy for the treatment of HCC. Song et al. demonstrated that lncRNA LINC00473-targeting shRNA impaired tumorigenesis by restoring miR-29a levels and perturb HCC metabolic regulation [58]. Similarly, Li et al. reported that shRNA-mediated silencing of circ-ZNF652 upregulates miR-29a to impede the glycolytic phenotype, resulting in the inhibition of tumor growth [57]. Gao et al. unveiled that siRNA-based silencing of LINC00473 boosts miR-29a levels to upregulate the Robo1 levels and accordingly the activation of PI3K/AKT/mTOR axis, thereby leading to ameliorate HCC tumor growth and metastasis [52]. 

## 9. Future Perspective

**Figure 7:**
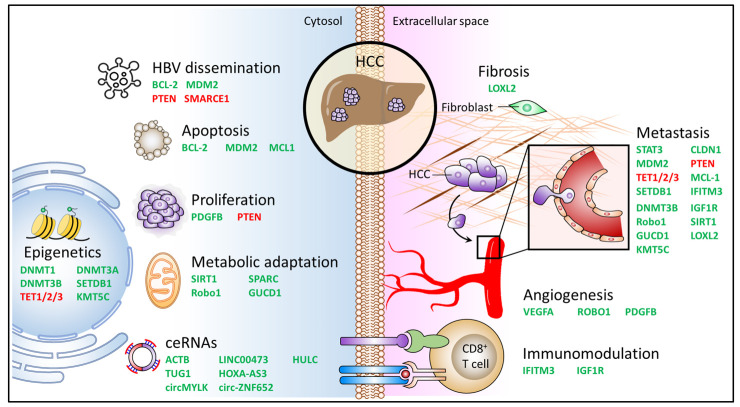
miR-29a targets in the progression and tumor microenvironment of HCC. Proposed model depicting targets of miR-29a involved in the molecular mechanisms and the formation of tumor microenvironment of HCC, including epigenetics, HBV dissemination, apoptosis, proliferation, metabolic regulation, competing endogenous RNAs (ceRNA), fibrosis, metastasis, angiogenesis, and immunomodulation. Targeted genes upon interacting with miR-29a conferring tumor-suppressing (green) or tumor-promoting (red) effect are summarized.

miR-29a is known to provide protective effect on liver damage and function as a critical gene which dictates hepatocarcinogenesis, epigenetic program, metabolic adaptation, ceRNAs, and TME components of HCC (Figure 7). Not like the presence of tri-uracil instability sequence (UUU) in miR-29b and miR-29c, the UCU sequence makes miR-29a more stable and sustainable. Its biological functions are predominantly involved in cancer progression and signaling pathway dictating tumorigenicity. Although a few reports showed miR-29a’s tumor-promotive role in HBV-involved tumor model, a large proportion of studies demonstrated its tumor-suppressive role in a pathway-centric activity. miR-29a’s role in restricting the stemness of CSCs seems to be a glimmer of hope to impede CSCs renewal and colonization, curb drug resistance, as well as alleviate side effects caused by chemotherapeutic agents. Its activity on inhibiting a cluster of methyltransferases and metabolic switches acts to restore the aberrant epigenetic profiles and metabolic program, respectively. In addition, miR-29a can remodel the fibrotic status of tumor circumstance and perturb molecular patterns to restrict the formation of pre-metastatic sites and the activation of metastasis. The role of miR-29a in targeting VEGF pathway can act to hamper the formation of vascular network and be applied to the development of anti-angiogenetic agents; its biological activity to counteract immunosuppression can pave a prospective path to the advance of immunotherapeutic booster. 

Numerous studies have revealed remarkable correlation of miR-29a with HCC diagnosis and outcome, therefore making miR-29a a promising biomarker for prediction and prognosis. However, the clinical significance of miR-29a in predicting outcome following a particular drug or drugs combination is still scarce and in need of further study. In terms of therapeutic use of miR-29a for HCC and other cancer types, more delicate pre-clinical studies, more specific and intelligently designed clinical trials are warranted. The identification of miR-29a-targeting ceRNAs recently shed light on more detail mechanisms of the sophisticated ncRNA field, rendering numerous evolving ceRNA-based therapeutic applications which aim at preventing HCC progression by way of miR-29a upregulation. A number of studies unveiled that compounds and small molecules relay their anti-HCC effect through the upregulation of miR-29a, implying the great potential of miR-29a as a candidate marker in drugs screening. However, the existing therapeutic modes of targeting miR-29a face the common challenges, including precise targeting of cancer cells, toxicity-free to other healthy tissues, and pharmaco-dynamic suitability such as preliminary biosafety and bio-distribution.

## Figures and Tables

**Table 1:** miR-29a and HCC carcinogenesis. List of the roles and mechanisms of miR-29a in carcinogenesis: stemness, apoptosis, proliferation, and HBV dissemination.

Role of miR-29a	Involved Mechanism	Used Model	Target Gene	Pathway	Outcome	Reference
−	Stemness, apoptosis	CSC	*BCL-2*	↑miR-29a →↓BCL-2	↓CSC self renewal ↑Sorafenib response ↓PDX growth	[46]
−	Apoptosis	SMMC7721 and Hep3B cells	*MDM2*	↑XPD→↑miR-29a →↓MDM2→↑p53	↓Proliferation ↓Migration	[45]
−	Apoptosis	Hep3B, HepG2 cells; Xenograft mice; Human HCC tissue	*MCL1*	Mutant ATCB →↓miR-29a →↑MCL1	↑Proliferation ↑Migration ↑Invasion ↑Tumor growth	[47]
−	Proliferation	SMMC7721 and Hep3B cells	*PDGFB*	↑XPD→↑miR-29a →↓PDGFB	↓Proliferation ↓Migration	[45]
+	HBsAg dissemination	HepG2 cell	*SMARCE1*	↑miR-29a →↓SMARCE1 →↑HBsAg	↑HBV replication	[44]
+	Proliferation	HepG2 and HepG2-X cell	*PTEN*	↑HBx →↑miR-29a →↓PTEN →↑p-AKT	↑Migration	[43]

“+”, pro-HCC effect; “−”, anti-HCC effect; “↑”, enhanced; “↓”, reduced; “→”, leading to a particular event; CLDN1, claudin1; CSC, cancer stem cells; HBsAg, hepatitis B surface antigen; HBV, hepatitis B virus; HBx, Hepatitis B virus X protein; MEG3, maternally expressed 3; PDGFB, platelet-derived growth factor subunit B; PTEN, phosphatase and tensin homolog; SMARCE1, SWI/SNF-related matrix-associated actin-dependent regulator of chromatin subfamily E member 1; XPD, Xeroderma pigmentosum D.

**Table 2:** miR-29a as an epigenetic modifier. List of the roles and mechanisms of miR-29a in epigenetic control.

Role of miR-29a	Involved Mechanism	Used Model	Target Gene	Pathway	Outcome	PMID
−	Epigenetic control	Hep3B and MHCC97L cells; Xenograft mice	*SETDB1*	↑miR-29a →↓SETDB1	↓Proliferation ↓Migration ↓Tumor growth ↓Tumor metastasis	[51]
−	Epigenetic control	HLE and SNU-475 cells; Xenograft mice; Human HCC tissue	*DNMT3A*	↑AFP→↑c-MYC →↓miR-29a →↑DNMT3A →↓tumor suppressor genes	AFP overexpression promotes a c-MYC-mediated miR-29a inhibition, leading to tumor growth and cellular proliferation, migration, and invasion.	[49]
−	Epigenetic control	HepG2, Huh-7, PLC/PRF-5, Hep-3B cell	*DNMT1, DNMT3B*	↑miR-29a →↓DNMT1, ↓DNMT3B →↑MEG3	↓Proliferation	[48]
+	Epigenetic control	HepG2, SMMC-7721, MHCC97H, and HCCLM3 cell; Xenograft mice	*TET1/2/3*	↑miR-29a →↓TET1/2/3 →↓SOCS1 →↑MM9	↓Apoptosis ↑Proliferation ↑Migration ↑Invasion ↑tumor growth ↑lung metastasis	[53]

“+”, pro-HCC effect; ”−”, anti-HCC effect; “↑”, enhanced; “↓”, reduced; “→”, leading to a particular event; AFP, α-fetoprotein; DNMT, DNA methyltransferase; MEG3, maternally expressed 3; MMP9, matrix metalloproteinase 9; PTEN, phosphatase and tensin homolog; SETDB1, SET domain, bifurcated 1; SOCS1, suppressor of cytokine signaling 1; STAT3, signal transducer and activator of transcription 3; TET, ten eleven translocation.

**Table 3:** Role and mechanism of miR-29a in metabolic adaptation. List of the roles and mechanisms of miR-29a in metabolic adaptation.

Role of miR-29a	Involved Mechanism	Used Model	Target Gene	Pathway	Outcome	PMID
−	Metabolic adaptation	HepG2, Huh7, HCCLM3 and SK-Hep-1 cells; Xenograft mice; Human HCC tissue	*Robo1*	↓LINC00473 →↑miR-29a →↓Robo1 →↓p-PI3K/p-AKT/p-mTOR	↓Proliferation ↓Migration ↓Invasion ↓Tumor growth ↓Tumor metastasis	[58]
−	Metabolic adaptation	MHCC97H cells	*SIRT1*	↑miR-29a→↓SIRT1	↓Proliferation ↓Cell cycle	[56]
−	Metabolic adaptation	97L and PLC cell	*SPARC*	↑miR-29a →↓SPARC →↓p-AKT →↓p-mTOR, ↓p-ERK	↓Proliferation	[55]

”–”, anti-HCC effect; “↑”, enhanced; “↓”, reduced; “→”, leading to a particular event; MMP9, matrix metalloproteinase 9; mammalian target of rapamycin; PI3K, phosphoinositide 3-kinase; Robo1, roundabout homolog 1; SETDB1, SET domain, bifurcated 1; SIRT1, sirtuin 1; SPARC, secreted multi-functional matricellular glycoprotein and rich in cysteine.

**Table 4:** ceRNA acts as miR-29a sponge to positively promote HCC progression. List of the recent insights into the roles of ceRNAs in tumorigenesis by sponging miR-29a.

ceRNA	Effect of ceRNA	Involved Mechanism	Used Model	Upregulated Gene	Pathway	Outcome	PMID
Mutant ATCB 3’UTR	+	Apoptosis	Hep3B, HepG2 cells; Xenograft mice; Human HCC tissue	*MCL1*	Mutant ATCB →↓miR-29a →↑MCL1	↑Proliferation ↑Migration ↑Invasion ↑Tumor growth	[47]
LINC00473	+	Metabolic regulation	HepG2, Huh7, HCCLM3 and SK-Hep-1 cells; Xenograft mice; Human HCC tissue	*ROBO1*	↑LINC00473 →↓miR-29a →↑Robo1 →↑p-PI3K/p-AKT/p-mTOR	↑Proliferation ↑Migration ↑Invasion ↑Tumor growth ↑Tumor metastasis	[58]
HULC	+	Epigenetic control	Hep3B, Huh7 cells	*SETDB1*	↑HULC →↓miR-29 →↑SETDB1	↑Proliferation	[60]
TUG1	+	Immunomodulation	MHCC-97H, HCC-LM3 cells	*IFITM3*	↑TUG →↓miR-29a →↑IFITM3	↑Proliferation↑Migration ↑Invasion↑Tumor growth	[61]
HOXA-AS3	+	Immunomodulation	Gastric cancer cells	*LTBR*	↑HOXA-AS3 →↓miR-29a →↑LTBR →↑NF-κB	↑Proliferation↑Migration ↑Invasion↑Tumor metastasis	[62]
circMYLK	+	Epigenetic control	MHCC-97H and HCC-LM3 cells; Xenograft mice; Human HCC tissue	*KMT5C*	↑circMYLK →↓miR-29a →↑KMT5C	↑Proliferation ↑Migration ↑Invasion ↑Tumor growth ↑Tumor metastasis	[52]
circ-ZNF652	+	Metabolic regulation	SNU-387 and Huh-7 cells; Xenograft mice; Human HCC tissue	*GUCD1*	↑circ-ZNF652 →↓miR-29a →↑GUCD1 →↑HK2	↑Glycolysis ↑Proliferation ↑Migration ↑Invasion ↑Tumor growth	[57]

“+”, tumor-promotive effect; “↑”, enhanced; “↓”, reduced; “→”, leading to a particular event; ATCB, beta actin; ceRNA, competing endogenous RNA; GUCD1, guanylyl cyclase domain containing 1; HK2, hexokinase 2; KMT5C, histone lysine N methyltransferase 5C; IFITM3, interferon-induced transmembrane protein 3; LTBR, lymphotoxin β receptor; MCL1, MCL1 apoptosis regulator, BCL2 family member; mTOR, mammalian target of rapamycin; ROBO1, roundabout homolog 1; SETDB1, SET domain, bifurcated 1; UTR, untranslated region.

**Table 5:** miR-29a as a regulator of tumor microenvironment. List of the roles and mechanisms of miR-29a in EMT, angiogenesis, and immunomodulation.

Role of miR-29a	Involved Mechanism	Used Model	Target Gene	Pathway	Outcome	PMID
−	Fibrosis	MHCC97L cells	*LOXL2*	↑miR-29a →↓LOXL2	↓Tissue stiffness ↓Tumor metastasis	[64]
−	Metastasis	SK-Hep-1 and Hep3B cells	*STAT3*	↑miR-29a →↓STAT3 →↓MMP-2/9	↓Proliferation ↓Migration ↓Invasion	[65]
−	Metastasis	HepG2 and HLE cells; Xenograft mice	*CLDN1*	↑miR-29a →↓CLDN1	↓Proliferation ↓Migration ↓Tumor growth	[66]
−	Angiogenesis	Glioma cells	*ROBO1*	↑miR-29a →↓ROBO1	↓Angiogenesis ↓Migration	[67]
−	Angiogenesis	EC	*PDGFB*	↑miR-29a →↓PDGFB	↓EC sprouting activity	[68]
−	Angiogenesis	Bladder cancer cells	*VEGFA*	circMYLK knockdown →↑miR-29a →↓VEGFA	↓Angiogenesis ↑Apoptosis ↓Proliferation ↓Migration	[69]
−	Immunomodulation	HCCLM3 cells	*IFITM3*	↑miR-29a→↓IFITM3	↑Apoptosis ↓Proliferation ↓Migration ↓Invasion	[70]
−	Immunomodulation	HepG2 cells	*IGF1R*	↑miR-29a →↓IGF1R (HepG2 cells) →↓CCL5 (CD8^+^ T cells)	↓Proliferation ↓Migration ↓Invasion ↑T cell recruitment	[71]

”−”, tumor-suppressive effect; “↑”, enhanced; “↓”, reduced; “→”, leading to a particular event; CCL5, C-C motif chemokine ligand 5; CLDN1, claudin1; EC, endothelial cells; IFITM3, interferon-induced transmembrane protein 3; IGF1R, insulin-like growth factor 1 receptor; MEG3, maternally expressed 3; LOXL2, lysyl oxidase like 2; MMP, matrix metalloproteinase; PDGFB, platelet derived growth factor subunit B; PTEN, phosphatase and tensin homolog; ROBO1, roundabout homolog 1; STAT3, signal transducer and activator of transcription 3; VEGFA, vascular endothelial growth factor A.

**Table 6:** Clinicopathological relevance of miR-29a in hepatocellular carcinoma (HCC).

Source	miR-29a Levels	Clinical Relevance	Reference
Tumor	↓	Biomarker for HCC; Predictor for lymph node metastasis, late TNM stage (III-IV)	[65]
Tumor	↓	Biomarker for HCC	[84]
Tumor	↓	Biomarker for HCC	[45]
Tumor	↓	Biomarker for HCC; Predictor for tumor size > 5 cm, vascular invasion, and poor DFS and OS	[56]
Tumor	↓	Biomarker for HCC; Predictor for tumor size > 5 cm, multifocal tumors, venous invasion, and poor OS	[70]
Tumor	↓	Biomarker for HCC	[85]
Tumor	↓	Biomarker for HCC; Predictor for poor OS	[66]
Tumor	↑	Biomarker for HCC; Predictor for poor OS	[53]
Tumor	↓	Inversely correlated with serum AFP of patients Predictor for poor prognosis	[49]
Tumor	↓	Biomarker for HCC	[55]
Tumor	↑	Predictor for early recurrence, and poor OS in HBV-related HCC after surgical resection	[86]
plasma	↓	Predictor for poor DFS and poor liver transplantation-free survival	[87]
Serum	↑	Differential biomarker for HBV-positive HCC; Predictor for poor OS and PFS	[88]
Serum	↑	Superior diagnostic factor to AFP; Predictor for small size, early-stage, AFP-negative HCC	[89]
Serum exosomes	↑	Diagnostic factor for HCC	[90]

“↑”, enhanced; “↓”, reduced; AFP, α-fetoprotein; DFS, disease-free survival; HBV, hepatitis B virus; OS, overall survival; PFS, progression-free survival.

**Table 7:** miR-29a-targeting approaches in experimental model.

Therapeutics	Biological Property	Model	Pathway	Outcome	Reference
Silencing of circ-ZNF652	shRNA-targeting circRNA	SNU-387 and Huh-7 cells; Xenograft mice	↓circ-ZNF652 →↑miR-29a →↓GUCD1 →↓HK2	↓Glycolysis ↓Proliferation ↓Migration ↓Invasion ↓Tumor growth	[57]
Silencing of circMYLK	siRNA-targeted circRNA	MHCC-97H and HCC-LM3 cells; Xenograft mice	↑miR-29a →↓KMT5C	↓Proliferation ↓Migration ↓Invasion ↓Tumor growth ↓Tumor metastasis	[52]
Silencing of LINC00473	shRNA-targeting lncRNA	HepG2, Huh7, HCCLM3 and SK-Hep-1 cells; Xenograft mice	↑miR-29a →↓Robo1 →↑p-PI3K/p-AKT/p-mTOR	↓Proliferation ↓Migration ↓Invasion ↓Tumor growth ↓Tumor metastasis	[58]
Xiaoai Jiedu Recipe (XJP)	Traditional Chinese medicine	SK-Hep-1 and Hep3B cells; Xenograft mice	↑miR-29a →↓STAT3 →↓MMP-2/9	↓Proliferation ↓Migration ↓Invasion ↓Tumor growth	[65]
Sevoflurane	Anesthetics	Huh-7 and HepG2 cells	↑miR-29a →↓DNMT3A	↑PTEN, ↓p-PI3K, ↓p-AKT ↑Apoptosis ↓Migration	[84]
Dendrosomal curcumin (DNC)	Natural phenol, delivered by dendrosome nanoparticle	Huh-7 and HepG2 cells	↑miR-29a/↑miR-185 →↓DNMT3A/↓DNMT3B/↓DNMT1 →↑MEG3	↓Cell viability	[91]
Arsenic trioxide	arsenic compound	HepG2 cell	↑miR-29a→↓*PPM1D*	↑Apoptosis	[92]
		Meta-analysis	NA	Potentiate therapeutic effect of TACE on: ↓AFP ↑one-year survival rate ↑life quality of PHC patients ↓chemotherapeutic side effects	[93]

“↑”, enhanced; “↓”, reduced; “→”, leading to a particular event; DNMT1, DNA methyltransferase 1; DNMT3A, DNA methyltransferase 3 alpha; DNMT3B, DNA methyltransferase 3 beta; KMT5C, histone lysine N methyltransferase 5C; MEG3, maternally expressed 3; MYC, MYC proto-oncogene, bHLH transcription factor; NA, not available; p-AKT, phospho-protein kinase B; p-PI3K, phosphorylated phosphoinositide 3-kinase; PPM1D, protein phosphatase Mg^2+^/Mn^2+^-dependent 1D; PTEN, phosphatase and tensin homolog; Robo1, roundabout homolog 1; TACE, transcatheter arterial chemoembolization.

## Data Availability

Data is contained within the article.
